# Grain Size Effect on the Hot Ductility of High-Nitrogen Austenitic Stainless Steel in the Presence of Precipitates

**DOI:** 10.3390/ma11061026

**Published:** 2018-06-15

**Authors:** Zhenhua Wang, Yong Wang, Chengming Wang

**Affiliations:** 1 School of Mechanical Engineering, Yanshan University, Qinhuangdao 066004, China; 528812958@stumail.ysu.edu.cn; 2 State Key Laboratory of Metastable Materials Science and Technology, Yanshan University, Qinhuangdao 066004, China; 3 HBIS Group Technology Research Institute, Shijiazhuang 050023, China; wangchengming@hbisco.com

**Keywords:** high-nitrogen austenitic stainless steel, hot ductility, precipitate, grain size, damage

## Abstract

Precipitation occurs easily during the hot forming of high-nitrogen austenitic stainless steels, which reduces their hot ductility significantly. The effect of grain size on the hot ductility of high-nitrogen austenitic stainless steel in the presence of precipitates was investigated. Different grain sizes of 18Mn18Cr0.5N steel specimens, with and without precipitates, were hot-tension tested. The precipitate morphology, fracture surface, and cracks were studied by scanning electron microscopy, transmission electron microscopy, and electron backscatter diffraction analysis. For the 18Mn18Cr0.5N steel, damage-formation strains of all grain-size specimens were reduced by the precipitates during the hot-tension test. Crack-formation sites were located at grain boundaries and were independent of the Taylor factor. A larger grain size resulted in an increased sensitivity of the fracture strain to precipitates. When the grain size was smaller than 51 μm, the fracture strain became insensitive to the precipitates. A method was suggested to mitigate surface cracking for metal materials with a high precipitation tendency.

## 1. Introduction

In high-nitrogen austenitic stainless steels (HNASs), in excess of 0.4 wt% N is added to replace expensive elemental Ni, which is usually contained in AISI 3xx series austenitic stainless steel [1]. N is an effective solution strengthener, an austenite stabilizer, and a corrosion-resistance enhancer [2,3,4]. Therefore, HNASs possess excellent mechanical, physical, and chemical properties. They are used widely in the energy, chemical, mechanical, and medical industries [5]. However, surface cracks form easily during the hot forming of HNASs, which restricts their application and development [6].

In previous studies, the influence of preheating temperature [6], strain rate [7], continuous cooling [8], grain size [9,10], and delta ferrite [11,12] on the hot ductility of HNASs was clarified in detail. However, these conditions differ from those encountered during the actual production of heavy-section HNAS components. In addition to the above factors, precipitates that form easily from 600 to 1050 °C [13,14] also affect the hot ductility of HNASs significantly [15]. During the hot forming of heavy-section HNAS components, where the surface may be cooled to a low temperature during forming [8,16], cracks form almost simultaneously with precipitation. However, the combined effect of precipitates and other factors remains unclear. A clarification of the effect of the influencing factors on the hot ductility in the presence of precipitates would have important industrial implications. 

In this study, 18Mn18Cr0.5N steel was selected as the model material. Specimens of different grain sizes with and without precipitates were hot-tension tested. The fracture surface, precipitate morphology, and cracks were examined. This study contributes to the development of the hot processing of HNAS components and other metal materials with a high precipitation tendency.

## 2. Materials and Methods 

Commercial 18Mn18Cr0.5N steel was melted in an induction furnace and then electroslag remelted. Its chemical composition was (wt%): 0.11 C, 18.46 Mn, 18.5 Cr, 0.54 N, 0.71 Si, 0.02 P, 0.01 S, 0.01 Al, and the balance was Fe. Samples (thickness: 30 mm; width: 30 mm; length: 100 mm) were cut from the remelted ingot and hot rolled at 1000 °C to a cumulative strain of approximately 1.6. The rolled samples were held at 1100 °C for 5 min, 1100 °C for 20 min, 1100 °C for 2 h, and 1200 °C for 3 h to achieve grain sizes of 28, 51, 106, and 177 μm, respectively. The grain morphology and grain-boundary character distribution of samples of different grain size are shown in [10,17]. 

Samples with different grain sizes were aged at 850 °C for 30 min to obtain precipitates. As reported in [18], after sensitization, the relative corrosion resistances of low-angle grain boundaries, Σ3 boundaries, and Σ9 boundaries were 100%, 95%, and 25%, respectively; other boundaries had no resistance to corrosion. That means that 100% low-angle grain boundaries, 95% Σ3 boundaries, and 25% Σ9 boundaries are resistant to precipitation. In 177, 106, 51, and 28-μm specimens, the total fraction of these three special boundaries are 53.0, 53.8, 57.4, and 49.0%, respectively [17]. Therefore, it is believed that grain size has no obvious effect on the fraction of grain boundary with precipitates.

Tension-test specimens (6-mm diameter × 120-mm length, 12-mm gage length) were cut from samples that contained precipitates. Specimens were preheated to 850 °C and then tension tested at a strain rate of 0.1 s^−1^ to fracture using a Gleeble 3800 simulator (Dynamic Systems Inc., Poestenkill, NY, USA). For comparison, specimens without precipitates were also tension tested under the same deformation conditions. To study the crack morphology, an additional specimen with a grain size of 177 μm and that contained precipitates was tension tested to a strain of 0.27 and was not broken.

The fracture surfaces of the broken specimens were examined by scanning electron microscopy (SEM) using a Hitachi S4800 instrument. The crack morphology in the unbroken specimen was studied using electron backscatter diffraction (EBSD) analysis with TSL-OIM-Analysis software. The precipitate morphology was studied by SEM and transmission electron microscopy (TEM) using a JEM-2010 instrument (JEOL Co., Ltd., Tokyo, Japan). 

## 3. Results and Discussion

### 3.1. Precipitate Morphology

**Figure 1:**
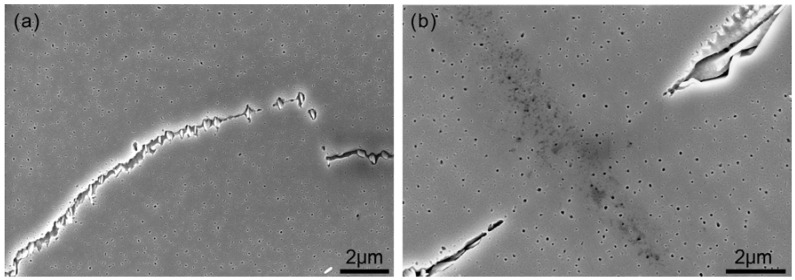
Typical SEM morphology of the precipitates: (**a**) granular and (**b**) lamellar.

Precipitates in specimens with different grain sizes were examined. The grain size had no significant effect on the precipitate morphology and distribution; precipitates were located discontinuously at grain boundaries. Figure 1 shows the typical SEM morphology of the precipitates. In Figure 1a, granular precipitates were discontinuous and located at grain boundaries. A groove or matrix existed between these particles. The groove existed because of precipitates that had been removed or because of a corroded matrix. In Figure 1b, the precipitates are lamellar and also discontinuous. The occurrence of precipitation at a section of the grain boundary depends on the grain-boundary type [18,19,20].

**Figure 2:**
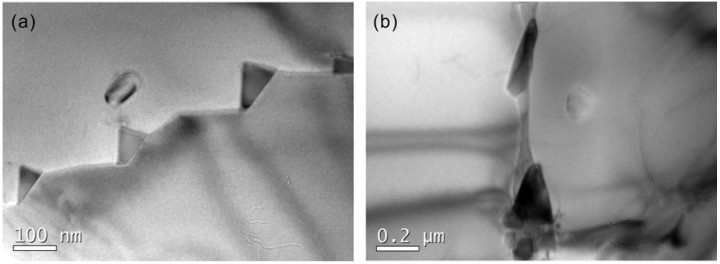
Typical TEM morphology of the precipitates: (**a**) granular and (**b**) lamellar.

Figure 2 shows the typical TEM morphology of the precipitates. In Figure 2a, granular precipitates form at a serrated grain boundary. Their shape and distribution indicate that during further aging, they will grow into discontinuous cellular precipitates [14,21]. Figure 2b shows the TEM morphology of a lamellar precipitate; its shape is similar to the precipitate in the upper corner of Figure 2b.

### 3.2. Flow Behaviors and Fracture Strains

**Figure 3:**
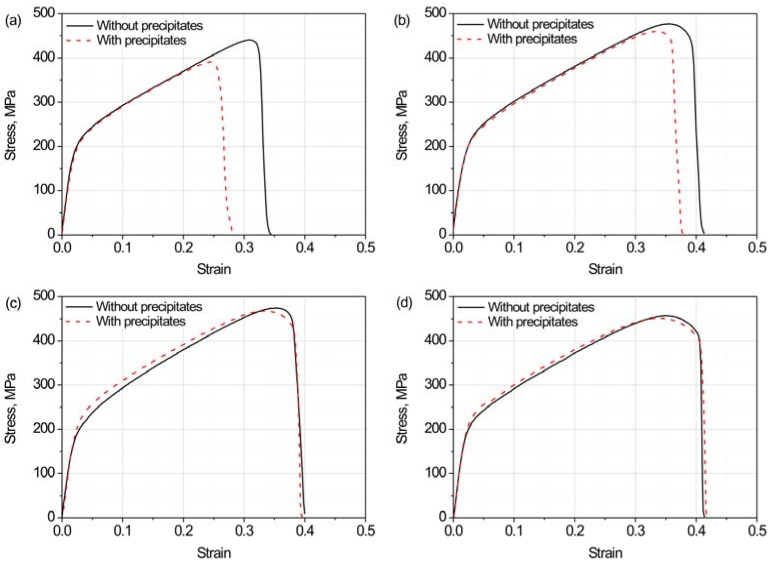
Flow curves of tension-tested specimens with different grain sizes (850 °C): (**a**) 177 μm; (**b**) 106 μm; (**c**) 51 μm; and (**d**) 28 μm.

Figure 3 shows flow curves of specimens with different grain sizes. The flow curves of specimens with precipitates are represented by a red dashed line and those without by a black solid line. In Figure 3a (177 μm), the flow curves of specimens with and without precipitates are almost coincident when the strain is below 0.2. Above 0.2, the strain-hardening rate is lower for the specimen with precipitates. At a strain of ~0.25, the flow stress of the specimen with precipitates decreases significantly. For the specimen without precipitates, the stress decreased at a larger strain of 0.32. Precipitates reduce the fracture strain under this grain-size condition. In Figure 3b (106 μm), the flow behaviors of specimens with and without precipitates are similar to the case in Figure 3a. However, the difference between specimens with and without precipitates in fracture strain is smaller in Figure 1b than in Figure 1a. In Figure 3c (51 μm) and d (28 μm), no obvious differences in flow stress and fracture strain exist between specimens with and without precipitates.

**Figure 4:**
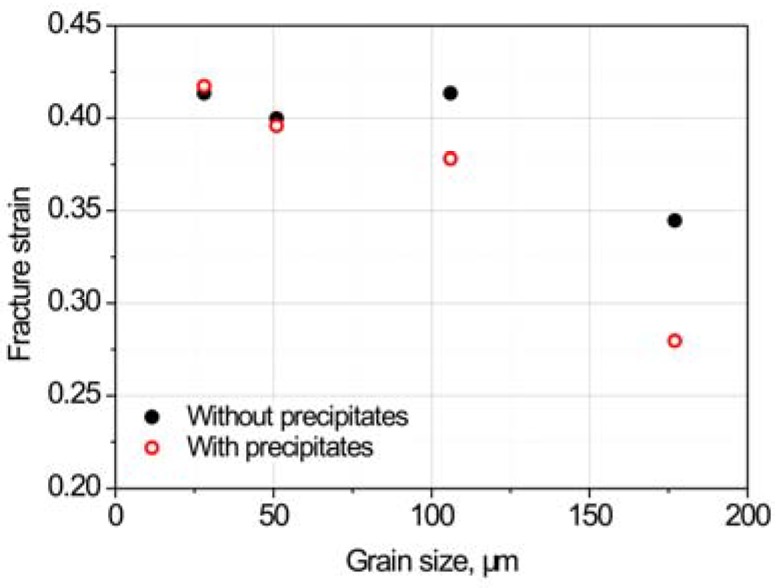
Fracture strains of specimens of different grain sizes with and without precipitates.

The fracture strains of specimens in Figure 3 were measured and are shown in Figure 4. The larger grain size results in an increased fracture strain sensitivity in the presence of precipitates. When the grain size is smaller than 51 μm, the fracture strain of the 18Mn18Cr0.5N steel becomes insensitive to the precipitates.

**Figure 5:**
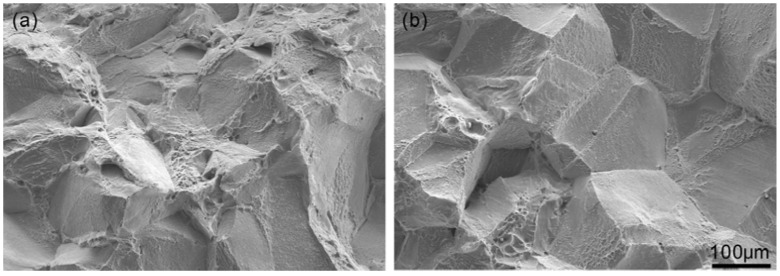
Fracture surface of specimens with a grain size of 177 μm: (**a**) without precipitates (**b**) with precipitates.

Figure 5 shows the fracture surface of the specimens with and without precipitates with a grain size of 177 μm. In Figure 5a (without precipitates), there is small volume fraction of ductile feature, i.e., dimples. In Figure 5b (with precipitates), the fracture is almost totally brittle; flat facets and straight grain edges are found easily. 

**Figure 6:**
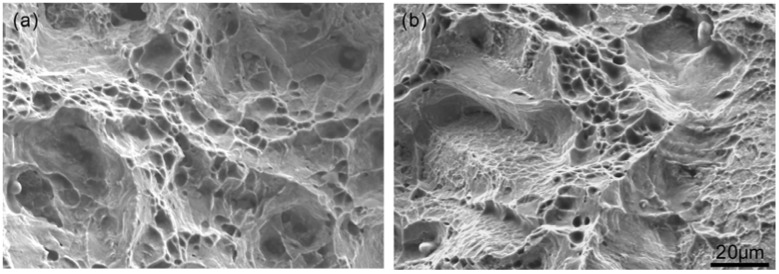
Fracture surface of specimens with a grain size of 51 μm: (**a**) without precipitates and (**b**) with precipitates.

Figure 6 shows the fracture surface of the specimens with and without precipitates with a grain size of 51 μm. A large number of dimples exist in Figure 6a and b, and no flat facets or straight grain edges were observed. Figure 5 and Figure 6 show that the fracture mechanism of large-grain-sized specimens is sensitive to the precipitates, whereas it is insensitive for fine-grained specimens. The phenomena presented in Figure 5 and Figure 6 are consistent with those in Figure 3 and Figure 4 and will be discussed in Section 3.3. 

### 3.3. Cracking and Damage Evolution

**Figure 7:**
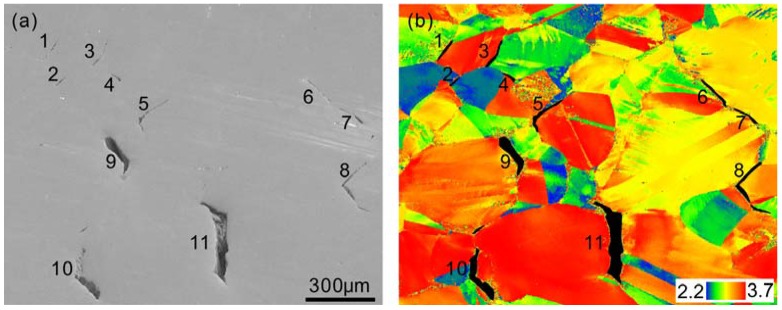
Morphology of cracks in a specimen with a grain size of 177 μm and with precipitates tension tested to a strain of 0.27: (**a**) SEM map and (**b**) Taylor factor map. Horizontal direction is the tensile direction.

A specimen with a grain size of 177 μm and with precipitates was tension tested to a strain of 0.27. The tension tested specimen was sectioned parallel to the tensile direction to examine the cracks. In Figure 7a, cracks are marked by numbers. Small cracks, 1–8, are newly formed cracks. Large cracks, 9–11, are developed cracks. The Taylor factor is used in the analysis of the plastic deformation of polycrystalline metals and implies the distribution of the grain orientations; grains can be classified into “hard” and “soft” based on their Taylor factors [22,23]. In the Taylor factor map (Figure 7b), cracks are shaded black. Cracks 1, 3, and 10 are each located between two grains with high and low Taylor factors. Crack 2 formed between two grains with low Taylor factors. Other cracks are each located mainly between two grains with high Taylor factors. The crack-formation sites are independent of the Taylor factors in Figure 7. However, in HNAS without precipitates, cracks formed mainly between grains with high Taylor factors [10]. Obviously, precipitates reduce the grain-boundary strength. In other words, the differences between the grain inner and grain boundary in strength are pronounced in the presence of precipitates. As a result, cracks are formed easily and become insensitive to the grain orientations.

**Figure 8:**
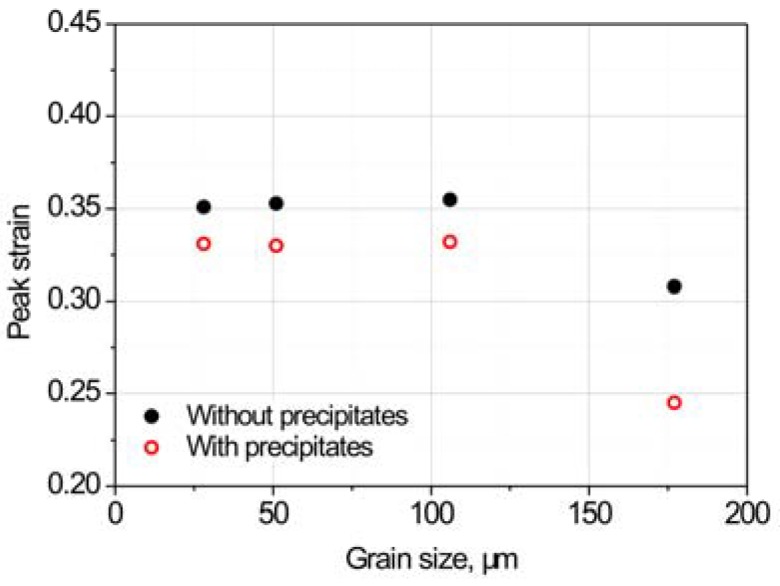
Peak strains of specimens of different grain sizes with and without precipitates.

Figure 8 shows the peak strains, i.e., the strains that correspond to the peak stresses, of specimens with different grain sizes. For all grain-size conditions, peak strains were sensitive to precipitates. During the tension test, damage such as cracks and cavities starts to form after the peak strain. The peak strain can be considered to be an initiation of damage formation. For all grain-size conditions, damage formation strains were sensitive to precipitates, which differs from the case of fracture strains that are insensitive to precipitates when the grain is smaller than 51 μm.

**Figure 9:**
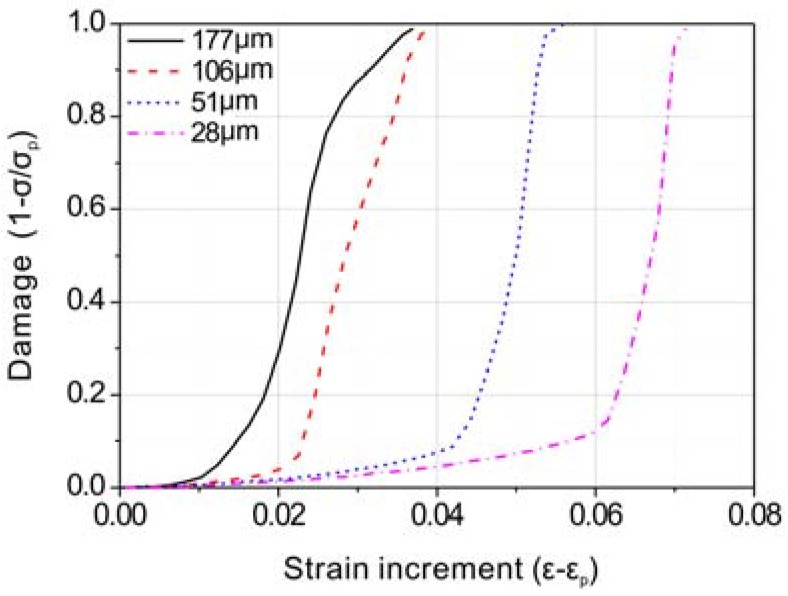
Damage evolution after peak strain for specimens with precipitates during tension test; ε_p_ is the peak strain.

The damage value at the peak strain is defined as 0. After the peak strain, the damage value increased with an increasing strain and a decreasing stress until the specimen was broken completely, where the damage value is defined as 1. The damage value between the peak strain and fracture strain is calculated from 1 − σ/σ_p_, where σ is the actual stress and σ_p_ is the peak stress. Figure 9 shows the calculated damage values after the peak strain for specimens with precipitates during the tension tests. The growth rate of the damage value is lower in the specimen with a finer grain size. This implies that, in the presence of precipitates, a finer-grained specimen can tolerate a larger increase in strain after the peak than a larger-grained specimen.

Precipitates reduce the grain-boundary strength and promote cracking (Figure 7). This is consistent with previous reports that a second phase at the grain boundaries induces a stress concentration around the boundary and leads to cavity formation [6,11,12,15]. Therefore, the peak strain, where damage commences, is sensitive to precipitation under all grain size conditions (Figure 4). 

For HNASs, precipitates were distributed discontinuously at some grain boundaries. During hot deformation, these sites with precipitates crack early. Grain-boundary segments with precipitates act as “pre-existing damage”. However, grain-boundary segments without precipitates still have high strength in the solution-treated state. Therefore, the reason that a finer grain size promotes grain rotation and a uniform strain distribution, and enhances dynamic recrystallization [9,10], also applies when discontinuous precipitates exists. This can explain why a finer-grained specimen can tolerate a larger further strain after the peak than a larger-grained specimen in the presence of precipitates.

Based on the above results, it is suggested that, for the hot forming of heavy-section components of HNASs such as heavy retaining rings, the grain size in the ingot surface layer should be minimized as much as possible during the early hot forming stage where the surface temperature is still high and precipitation has not occurred yet. In the latter forming stage, the temperature of the ingot surface falls into the precipitation temperature range and precipitates appear. At this point, if the grain size is large, the surface cracks will develop rapidly into the inside of the ingot because a coarse grain size cannot tolerate any further strain after damage formation. In contrast, if the grain size is sufficiently small, the surface cracks remain small and do not link together; it is difficult for small surface cracks to propagate into the ingot inner during the ensuing deformation process. In addition to HNASs, many high-alloy steels and nickel alloys have a high precipitation tendency. During the hot forming of heavy-section components, the method of compressing the ingot surface quickly and lightly in the early stage of hot forming, which refines the grain size in the surface layer, can be used to mitigate surface cracking caused by precipitation.

## 4. Conclusions

For 18Mn18Cr0.5N steel, damage-formation strains of all grain-size specimens are reduced by precipitates during hot tension tests. The crack-formation sites are located at grain boundaries and are independent of the Taylor factor. The damage growth rate is lower in specimens with a finer grain size. When the grain size is larger than 106 μm, precipitates reduce the fracture strain. A larger grain size results in an increased sensitivity of the fracture strain to precipitates. When the grain size is smaller than 51 μm, the fracture strain becomes insensitive to the precipitates. During the hot forming of heavy-section components, the method of compressing the ingot surface quickly and lightly in the early stage of hot forming can be used to mitigate surface cracking caused by precipitation.
